# Cultivable Skin Mycobiota of Healthy and Diseased Blind Cave Salamander (*Proteus anguinus*)

**DOI:** 10.3389/fmicb.2022.926558

**Published:** 2022-07-14

**Authors:** Polona Zalar, Ana Gubenšek, Cene Gostincar, Rok Kostanjšek, Lilijana Bizjak-Mali, Nina Gunde-Cimerman

**Affiliations:** ^1^Chair of Molecular Genetics and Biology of Microorganisms, Department of Biology, Biotechnical Faculty, University of Ljubljana, Ljubljana, Slovenia; ^2^Chair of Zoology, Department of Biology, Biotechnical Faculty, University of Ljubljana, Ljubljana, Slovenia

**Keywords:** *Proteus anguinus*, olm, fungi, opportunistic pathogens, *Saprolegnia*

## Abstract

*Proteus anguinus* is a neotenic cave salamander, endemic to the Dinaric Karst and a symbol of world natural heritage. It is classified as “vulnerable” by the International Union for Conservation of Nature (IUCN) and is one of the EU priority species in need of strict protection. Due to inaccessibility of their natural underground habitat, scientific studies of the olm have been conducted mainly in captivity, where the amphibians are particularly susceptible to opportunistic microbial infections. In this report, we focused on the diversity of cultivable commensal fungi isolated from the skin of asymptomatic and symptomatic animals obtained from nature (20 specimens) and captivity (22 specimens), as well as from underground water of two karstic caves by direct water filtration and by exposure of keratin-based microbial baits and subsequent isolation from them. In total 244 fungal isolates were recovered from the animals and additional 153 isolates were obtained from water samples. Together, these isolates represented 87 genera and 166 species. Symptomatic animals were colonized by a variety of fungal species, most of them represented by a single isolate, including genera known for their involvement in chromomycosis, phaeohyphomycosis and zygomycosis in amphibians: *Acremonium*, *Aspergillus*, *Cladosporium*, *Exophiala*, *Fusarium*, *Mucor*, *Ochroconis*, *Phialophora* and *Penicillium*. One symptomatic specimen sampled from nature was infected by the oomycete *Saprolegnia parasitica*, the known causative agent of saprolegniosis. This is the first comprehensive report on cultivable skin mycobiome of this unique amphibian in nature and in captivity, with an emphasis on potentially pathogenic fungi and oomycetes.

## Introduction

Cave salamander *Proteus anguinus*, also known as the olm, is a symbol of world natural heritage, a flagship species of the subterranean environment and a Slovenian national symbol. Olm is the longest-living amphibian, the largest troglobiotic tetrapod in the World and the only European troglobiotic vertebrate. Original description dates in Laurenti (1768). Since the discovery of pigmented form (Sket and Arntzen, 1994), two subspecies are known: the originally described and more spread non-pigmented variant *P. anguinus anguinus* (Paa), and the black olm or *P. anguinus parkelj* (Pap). Olm belongs to the family Proteidae, an ancient group of aquatic salamanders with only two genera, the European *Proteus* (with one currently recognized species) and the North American genus *Necturus* (with five currently recognized species). While *Necturus* lives in surface water, *Proteus* is endemic to the subterranean waters of the Dinaric Karst of souterneast Europe, with most known localities in Slovenia (Sket, 1997; Gorički et al., 2017). Its major troglobiotic adaptations include depigmented skin, degenerated eyes and elongated body parts, high tolerance to anoxia, slow metabolism, and unusual resistance to starvation. Since olms are also neotenic, they retain certain larval characteristics through their life, including external gills, gill slits and skin morphology (Bulog et al., 2000; Voituron et al., 2011; McGaugh et al., 2020; Kostanjšek et al., 2021a).

The olm is one of the EU priority species in need of strict protection (Vörös et al., 2017), listed in the EU Habitats Directive and Bern and Ramsar Conventions, and is classified as “vulnerable” by the International Union for Conservation of Nature “(IUCN Red)” (Arntzen et al., 2009) and EDGE lists (Safi et al., 2013).^1^ In Slovenia it is protected by national legislation since 1922.

As an endemic and neotenic species with a narrow ecological niche, olm is highly susceptible to infections (Heard et al., 2013), especially to pathogens with a high mortality potential for urodelans (Price et al., 2016; Spitzen-van der Sluijs et al., 2016) and amphibians in general. According to the Global Amphibian Assessment (GAA), more than 40% of amphibian species are in decline, while an additional 32% are threatened (Latney and Klaphake, 2013). The main microbial threats to amphibian diversity are the fungal disease chytridiomycosis, caused by the fungi *Batrachochytrium dendrobatidis* and *B. salamandrivorans* (Scheele et al., 2019), and ranaviruses (Price et al., 2016; Spitzen-van der Sluijs et al., 2016). Both chytrids infect many amphibian species across Europe, including countries neighboring Slovenia (Fisher and Garner, 2020). During the first monitoring study performed in Slovenia between 2015 and 2019, we collected swab samples from 173 live amphibians of 22 species, from 53 natural sites across Slovenia and from 41 captive amphibians. Sampling also included 70 olm individuals from five wild populations in Slovenia and 18 captive specimens. All samples were analyzed by real-time quantitative polymerase chain reaction (qPCR) for the presence of *B. salamandrivorans* (Blooi et al., 2013) and ranaviruses (Leung et al., 2017). We identified a single infection with *B. dendrobatidis* on an edible frog (*Pelophylax* kl. *esculentus*) from a natural habitat and no infections with *B. salamandrivorans* or ranaviruses of other amphibians sampled, including the olm (Kostanjšek et al., 2021b).

Other important fungal infections that contribute to global amphibian declines (Latney and Klaphake, 2013) include chromomycosis, phaeohyphomycosis, zygomycosis, and saprolegniosis, the latter caused by water molds (Oomycota, Stramenopiles). Due to the unique physiology and ecology of amphibians, water is one of the main vectors for transmission of these pathogens (Robert et al., 2011). Although it is known that stressed animals in captivity can become infected *via* traumatized gills or skin and through contact with contaminated water (Seyedmousavi et al., 2013), only three reports on fungal infections of olms in captivity have been published so far (Kogej, 1999; Bizjak-Mali et al., 2018; Lukač et al., 2019; Li et al., 2020), and no data exist on fungal infections of the olm in its natural underground habitats.

The focus of this study was isolation and identification of cultivable fungi from the skin of olm specimens inhabiting underground water of five karstic caves, representing different Slovenian natural populations of olm (Sket, 1997; Gorički et al., 2017) and from three vivaria where they were kept in captivity. Commensal fungi were isolated both from the skin of healthy animals and from animals with visible symptoms of disease, as well as from microbial baits exposed in water of three selected karstic caves, and by water filtration from two locations. Results of this study provide the first comprehensive insight into cultivable skin mycobiota of the olm and into its exposure and susceptibility to fungi populating underground karstic water environment, with emphasis on potential pathogens.

## Materials and Methods

### Sampling Sites

**FIGURE 1:**
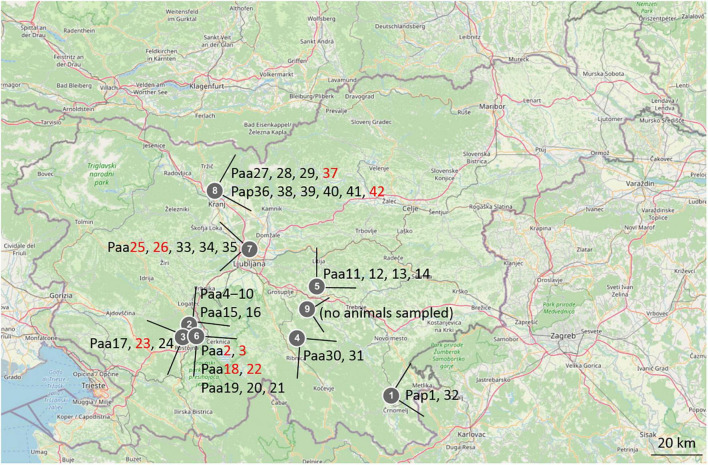
Map of Slovenia with marked sampling locations and sampled olm specimens. 1-Jelševnik, 2-Planinska jama, 3-Èrna jama, 4-Kompoljska jama, 5-Vir pri Stièni, 6-Vivarium 1, 7-Vivarium 2, 8-Vivarium 3, 9-Krška jama. The labels of animals (Paa, *Poteus anguinus anguinus*; Pap, *Proteus anguinus parkelj*) are written next to locations, in black for asymptomatic, and in red for symptomatic animals.

Animals of *Proteus anguinus* have been sampled in five different karstic caves in Slovenia: Planinska jama and Črna jama, both part of Postojna–Planina cave system in southwestern Slovenia, Vir pri Stični, Kompoljska jama and Jelševnik in southeastern Slovenia (Figure 1), as well as three different sites (vivaria) where olm was kept in captivity. Water from Vivarium 1 originated from the Postojna–Planina cave system, water in Vivarium 2 was dechlorinated tap water, while water in Vivarium 3 originated from the vicinity of Kranj SW Slovenia. Additional water samples were taken in Krška jama, where no olms were found. In total 42 skin swab samples were collected from 42 animals (20 in the wild, 22 in captivity) over the period of 3 years (2017–2019). Nine of the swabs were taken from symptomatic animals, one of them was from the natural habitat and the other eight from captivity. Sampling sites and olm specimens are presented in Figure 1 and in Supplementary Table 1.

### Sampling of Animals and Environment

#### Animals

Animals were collected under permit 35601-27/2021-8 issued by the Ministry of the Environment and Spatial Planning of the Republic of Slovenia and Slovenian Agency for the Environment. Skin of asymptomatic olm specimens was sampled for the presence and identification of commensal fungi. The specimens were handled with fresh sterile gloves and were rinsed with 100 ml of sterile water prior to sampling to ensure that the skin sample primarily included skin-associated fungi. Sampling was non-destructive using sterile swabs (4N6 FLOQSwabs^®^, COPAN) with plastic shafts following the standard procedures established for amphibians (Boyle et al., 2004; McKenzie and Peterson, 2012). Immediately after sampling the swabs were placed into sterile transport Amies liquid at 4°C and processed within 24 h after sampling. Animals were released immediately after sampling at the site where they were captured. Symptomatic specimens were swab-sampled only in regions with visible signs of disease. In order to control a potential disease, spread and to get more information about the pathology, they were then euthanized by prolonged immersion in 1.0% tricaine methanesulfonate solution (MS222, Sigma Chemical) buffered with 0.2% sodium bicarbonate (pH 7) and were submitted for necropsy. The carcass of these specimens and the isolated organs were fixed in 10% buffered formalin, rinsed in tap water, and then stored in 70% ethanol. Gross pathological changes were observed and documented using the MZFLIII dissecting stereomicroscope (Leica) equipped with the DFC 425C digital camera (Leica) and LAS software (V4.0) (Leica).

#### Environment

**FIGURE 2:**
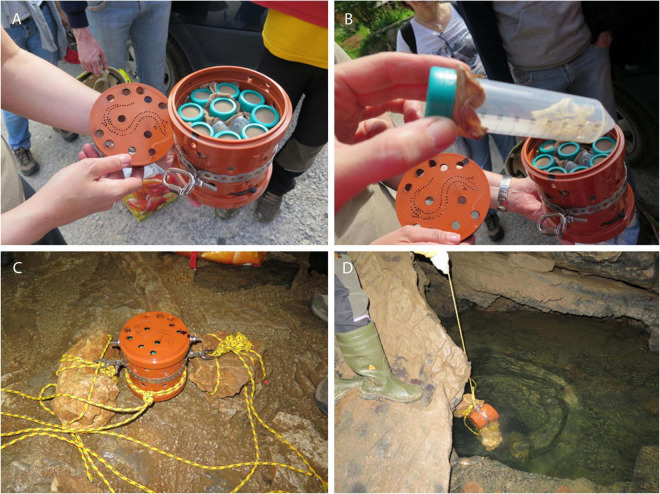
Baits placed in Falcon tubes, closed with nylon textile, protected by perforate plastic container, firmly fixed into cave water by ropes.

At selected sampling sites in caves (Jelševnik, Krška jama, Planinska jama), keratin baits (moulten snake skin, chicken feathers), and dialysis tubes filled with water agar, all separately sterilized by autoclaving in 50 ml Falcon tubes and covered with nylon fabric, were placed into perforated plastic containers, and exposed to the cave water by fixation with ropes (Figure 2). Baits were retrieved after 1, 3, 6, 12 and 15 months and checked for the presence of fungi. Water was collected in sterile containers at the point of animal capture, and in aliquots of 100 mL filtered through 0.45 μm membrane filters (Millipore) using a vacuum.

### Isolation and Characterization of Fungi and Oomycetes

#### Animals

Fungi and oomycetes were isolated from the skin swabs of asymptomatic and symptomatic olms from nature and captivity, sampled as described above. Tubes with swabs and transport liquid were vigorously shaken in order to release the material from the swabs; 30–50 μl of transport liquid was pipetted on the surface of DRBC with chloramphenicol (50 mg/L), SabG, and TGhL with a mixture of penicillin/streptomycin (penicillin-G: 200 mg/L, streptomycin: 400 mg/L). In addition, the same amount of Amies, Copan transport liquid, was placed into a Petri dish with cooked hemp seeds (100°C, 20 min) and sterile distilled water was added up to 20 ml.

#### Environment

Fungi and oomycetes were also isolated from microbial baits incubated in water and from cave water samples. Baits were cut in approx. 1 × 1 cm^2^ pieces and placed onto the surface of all above listed culture media. After environmental water filtration, filters were placed onto the following culture media: Dichloran Rose Bengal Chloramphenicol agar (DRBC; Biolife), Sabouraud’s dextrose agar (SabD; Biolife), malt extract agar (MEA), tryptone gelatine hydrolyzate lactose agar (TGhL; Longcore et al., 1999).

All culture media were inoculated in duplicates; one set was incubated at 15°C (close to the temperature of cave environment, appropriate for *B. salamandrivorans*) and the other at 20°C (appropriate for *B. dendrobatidis*) until visible growth. Fungi were isolated in pure cultures and deposited in the Ex Culture Collection of the Infrastructural Centre Mycosmo, MRIC UL, Slovenia,^2^ at the Department of Biology, Biotechnical Faculty, University of Ljubljana. All isolated fungi were identified to the genus or species level by their morphology and by sequencing the molecular taxonomic markers as described below. Microscopic characters were observed with Nomarski interference contrast optics on Olympus BX-51 microscope, and micrographs were recorded with DP72 camera (Olympus).

### Molecular Identification of Isolates

Genomic DNA of yeast isolates was extracted using the PrepMan Ultra reagent (Applied Biosystems) according to the manufacturer instructions. DNA of filamentous fungi was extracted after mechanical lysis in CTAB buffer according to the protocol described by Gerrits van den Ende and de Hoog (1999). DNA regions/genes used for identification were internal transcribed spacers 1 and 2 including the 5.8S rDNA (ITS), partial large ribosomal subunit rDNA including its D1/D2 domains (LSU) for the majority of isolates, partial sequences of genes encoding for actin (*act*) for genus *Cladosporium*, translation elongation factor one alpha (EF-1α) for genera *Trichoderma* and *Fusarium*, and β-tubulin (*benA*) for genera *Aspergillus* and *Penicillium*. These were PCR amplified and Sanger sequenced with the following primer sets: ITS1/ITS4 and NL1/NL4 (White et al., 1990), ACT-512F/ACT-738R (Carbone and Kohn, 1999), EF1-983F/EF1-2218R (Rehner and Buckley, 2005), Ben2f (Hubka and Kolarik, 2012)/Bt2b (Glass and Donaldson, 1995). After sequencing the sequences of the most similar type strains and other taxonomically important reference strains were retrieved from the non-redundant GenBank nucleotide database with the blast algorithm (Altschul et al., 1990) and aligned with sequences of olm isolates, followed by phylogenetic analyses with maximum likelihood method as implemented in Mega7, version 7 (Kumar et al., 2016). All DNA sequences from the representative strains from this study were deposited in the GenBank database: ON261225–ON261330, ON312944–ON312998 (ITS rDNA), ON777803–ON777808 (actin), ON777793–ON777802 (beta tubulin), ON804224–ON804232 (translation elongation factor 1-alpha).

### Statistical Methods

To identify potential connections between environmental variables and microbial diversity, machine learning methods were used to analyze the data. All analyses have been performed in the environment R and Microsoft Excel, 2016.

Hierarchical clustering was employed to determine the similarity among samples, using function “hclust()” from the package “stats” v3.6.1, which is part of the base R.

Correlation of occurrence of fungal species was obtained by the function “cor()” from the package “stats” v3.6.1. Graphical images were produced with the function “pheatmap()” from the package “pheatmap” v1.0.12 (Kolde, 2019). Species cooccurrence was investigated with the package “cooccur” v1.3 (Griffith et al., 2016) in R. R package “randomForest” (Liaw and Wiener, 2002) was used for the construction of decision trees as a means to identify fungal taxa (based on their presence/absence) with good predictive power of different environmental variables (e.g., animal health or captivity). We have calculated 10001 trees at each condition studied. Visualization of taxonomic units was made by software package ggplot2 (Wickham, 2016).

## Results

### Sampling of Healthy and Symptomatic Animals

Over a 2-year period, a total of 42 animals were sampled in five karstic caves and at three locations where animals are held in long-term captivity (Figure 1). Thirty-three swab samples (78.6%) were from healthy, asymptomatic animals, out of which 19 were from the natural subterranean environment (57.6%) and 14 were from captivity (42.4%). Nine symptomatic animals are briefly described in Table 1. Eight were from captivity and one from the natural environment of the Postojna-Planina cave system (from Črna jama cave).

**TABLE 1:** Symptoms of visibly diseased (symptomatic) animals.

Animal	Captivity (C)/Nature (N) and location	Symptoms
Paa2	C (Vivarium 1)	Extensive and recurring edema of body cavity, yellow nodular formations on kidneys and mottled lesions on internal organs
Paa3	C (Vivarium 1)	Lethargic, underweight
Paa18	C (Vivarium 1)	Moldy overgrowth on the detached limb
Paa22*	C (Vivarium 1)	Visible mycelium on the limb
Paa23	N (Črna jama, Planina-Postojna cave system)	Extensive edema of body cavity, yellow nodular formations on internal organs
Paa25*	C (Vivarium 2)	Lethargic, visible mycelia on the head and limbs
Paa26	C (Vivarium 2)	Visible moldy overgrowth on the body surface
Paa37	C (Vivarium 3)	Edemas in pectoral and pelvis region
Paa42	C (Vivarium 3)	Lethargic

*Animals originated from the Planina-Postojna cave system. They showed symptoms after 1 month in captivity.

### Fungal Diversity From Asymptomatic Animals

Total number of fungal isolates from all animals was 244. These isolates represent 73 different genera and 115 species. The complete list of isolated fungi from all animals is presented in Supplementary Table 2, and the list of fungal species isolated from at least two animals in Table 2. The highest diversity of fungi was determined for 14 asymptomatic captive animals. Fungi that were isolated exclusively from these animals belong to 39 different genera and 60 different species. On average, 9.5 strains and 7.4 different species were isolated per animal (3 to 22 strains and 1 to 18 different species per animal). The majority of species were represented by only one or two isolates. Species that were represented with at least 3 and up to 7 isolates were: *Acremonium* sp. (nov.), *Apiotrichum laibachii*, *Ap. porosum*, *Candida saitoana*, *C. sake*, *Cyphellophora olivacea*, *Dipodascus geotrichum*, *Exophiala castellanii*, *Penicillium roseopurpureum*, *P. roseopurpureum*, *Pseudogymnoascus* spp., *Samsoniella hepiali*, and unidentified isolates belonging to *Rutstroemiaceae*.

**TABLE 2:** List of fungal species isolated from at least two sampled olm specimens: asymptomatic animals (A), symptomatic animals (S), natural cave environment (N), captivity (C), number of olm specimens from which fungal strains were isolated (P).

Taxon name	A	S	N	C	P	Olm specimen
*Acremonium* sp. (nov.)*	+	+		+	6	Paa20, Paa21, **Paa26**, Paa29, Pap36, Pap39
Apiotrichum laibachii**,^Y^	+			+	3	Paa33, Paa35, Paa41
Aspergillus creber*	+	+	+	+	2	Paa20, **Paa22**
Aspergillus jensenii*	+	+		+	2	Paa28, **Paa42**
Bjerkandera adusta**	+		+	+	2	Paa15, Paa41
Candida vartiovaarae*,^Y^	+	+		+	2	**Paa26**, Paa35
Cladosporium allicinum*		+	+	+	2	**Paa2**, **Paa23**
Cladosporium neolangeronii*	+	+	+	+	3	Paa15, Paa20, Paa29
Cladosporium pseudocladosporioides*	+	+	+	+	3	Paa2, Paa7, Paa41
Cyphellophora olivacea*	+			+	2	Paa20, Paa21
*Cystofilobasidium* sp. (nov.)**,^Y^	+		+		3	Paa30, Paa31, Paa32
Debaryomyces hansenii*,^Y^	+	+	+	+	4	Paa32, Pap38, Pap39, Paa40
Dipodascus geotrichum*,^Y^	+			+	2	Paa35, Paa41
Exophiala castellanii*,^*BY*^	+			+	2	Paa20, Paa21
Lecanicillium coprophilum*	+	+		+	4	Paa29, Pap36, **Paa37**, Pap39
Mucor circinelloides***	+			+	2	Paa33, Paa35
Mucor racemosus***	+			+	2	Paa33, Paa35
Parengyodontium album*	+	+	+	+	2	Paa11, Paa27
Penicillium atrosanguineum*	+			+	2	Pap38, Pap39
Penicillium brevicompactum*	+	+	+	+	2	**Paa2**, Paa32
Penicillium chrysogenum*	+	+	+	+	5	**Paa2**, Paa20, **Paa18**, Paa27, Paa28, Paa29
Penicillium citreonigrum*	+			+	2	Pap38, Pap39
Penicillium roseopurpureum*	+			+	3	Paa20, Pap38, Pap39
*Pseudogymnoascus* sp.*	+			+	4	Paa20, Paa21, Paa27, Paa28
Pyrenochaetopsis leptospora*	+	+	+		2	Pap1, **Paa23**
Samsoniella hepiali*	+			+	2	Pap36, Paa41
Saprolegnia parasitica*****		+	+		2	**Paa22**, **Paa25**
Trichoderma simmonsii*	+	+	+	+	2	**Paa3**, Paa4
Trichoderma viride*	+			+	2	Paa33, Paa35

+Isolation of fungal species from a particular source. *****Affiliation to the main fungal phyla, *Ascomycota, **Basidiomycota, ***Mucoromycota, ****Chytridiomycota, ***** Oomycota; ^Y^: yeast; ^BY^: – black yeast; Paa, Poteus anguinus anguinus; Pap, Proteus anguinus parkelj. Labels of symptomatic animals are written in bold and underlined.

**FIGURE 3:**
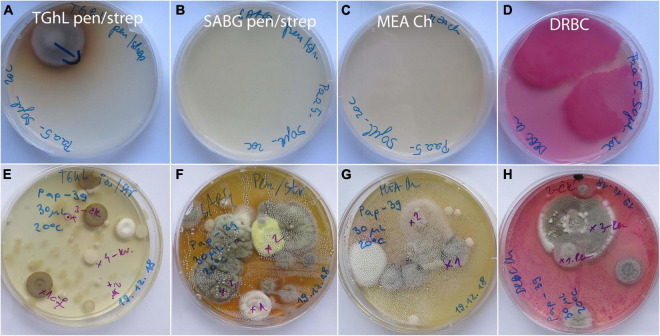
Primary isolation plates after 14 days of incubation at 20°C with skin surface swab samples of asymptomatic *Proteus anguinus*. **(A–D)** Paa5 from wild; **(E–H)** Pap39 from captivity (Vivarium 2).

On 19 asymptomatic animals sampled in the wild, fungal diversity was much lower (Supplementary Table 2). In 6 animals, no fungal strains were obtained at all, while on the remaining 13 animals, 12 different species from 11 genera were found. On average, 1.3 isolates were obtained per animal (1 to 6 strains, 1 to 4 different species per animal). Again, the frequency of most species was 1 to 2 strains per animal (Supplementary Table 2). Only two species were represented by six (*Cystofilobasidium* aff. *macerans*) and five strains (*Tausonia pullulans*), respectively. With the exception of one species (*Bjerkandera adusta*), there was no overlap in the diversity of fungi from asymptomatic animals in nature and captivity. Examples of culture media with swab samples from asymptomatic animals from wild and captivity, incubated for 14 days at 20°C, are presented on Figure 3.

### Fungal Diversity From Symptomatic Animals

**FIGURE 4:**
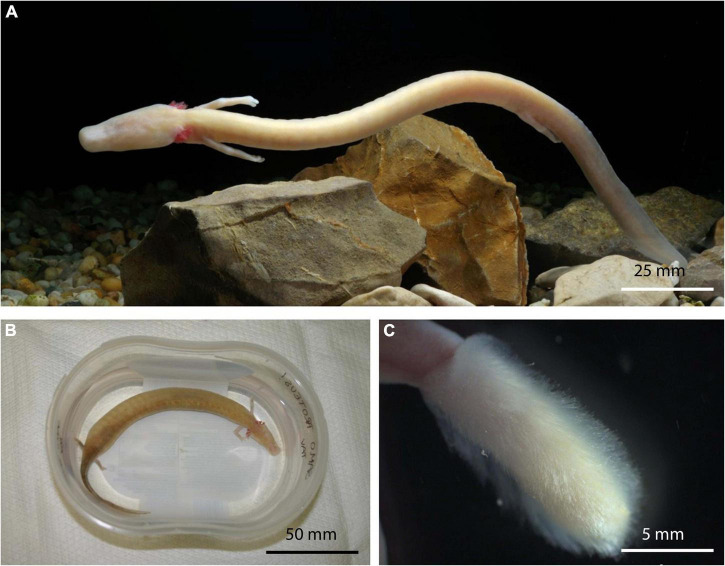
Healthy asymptomatic olm, *Proteus anguinus* from captivity **(A)**. Specimen Paa23 with extensive edema throughout body cavity **(B)**. Dense, boot-resembling *Sporolegnia* overgrowth on hind limb of symptomatic animal Paa22 **(C)**.

Fungi were isolated also from animals (Figure 4A) with very different symptoms. Summarized symptoms of animals are presented in Table 1.

Based on the most common pathologies, the symptomatic animals were assigned to three groups: (i) edema, (ii) visible fungal overgrowth, and (iii) lethargy.

(i) Edema is the most prominent morphologic pathology in olms. They occasionally grow to extent of impaired movement and disfigurement of symptomatic specimens. The first animal in this group (specimen Paa2) developed an extensive edema throughout the body cavity after being held in captivity for over 10 years. After removal of 22 ml of fluid from the body cavity, the edema recurred after 3 months. The recurrence of edema was accompanied by bleeding into the body cavity and lethargy of the animal. Autopsy of the euthanized specimen revealed yellow nodular formations on the kidneys and white mottled lesions on the internal organs. Two fungal genera were isolated: melanized filamentous *Cladosporium allicinum, Cl. pseudocladosporioides*, and filamentous *Penicillium brevicompactum*, and *P. chrysogenum* (Table 2). Similar pathology was observed in specimen Paa23 captured in the natural environment of the Postojna-Planina cave system. Again, the pronounced edema extended over the entire body cavity (Figure 4B) and autopsy revealed yellow nodular formations on the liver and kidneys. The specimen was colonized by 15 different genera comprising 15 species (data shown in Table 2 and in Supplementary Table 2). Among them three melanized species (*Cladosporium allicinum*, *Cadophora melinii*, and *Phialocephala glacialis*) are recognized as potential causative agents of phaeohyphomycosis. Other isolated species were *Cystobasidium minutum*, *Juxtiphoma eupyrena*, *Plectosphaerella plurivora*, *Pyrenochaetopsis leptospora*, *Sistotrema brinkmannii, Talaromyces kabodanensis, Truncatella angustata*, and *Xylodon flaviporus*, potentially new species of genera *Paracremonium* and *Paraphoma*, and yet unidentified species belonging to Pleosporales and Helotiales. The edemas of a third specimen in this group (specimen Paa37) were localized in the pectoral and pelvic regions. Specimen developed symptoms after being kept in captivity for several years and only a single fungal species was isolated from the animal: *Lecanicillium coprophilum.*

(ii) The group of symptomatic animals with visible fungal overgrowth includes four specimens. The first specimen, Paa18, was a 2-year-old juvenile bred in Postojna cave aquaria. The specimen lost its hind limb after a fight with its sibling. Direct microscopy revealed coenocytic hyphae and sporangia typical for *Cunninghamella*, which was not isolated in pure culture. Isolated fungi from hyphal overgrowth of the injured limb included three fungal species: *Penicillium chrysogenum, Sydowia polyspora* and *Trichoderma citrinum*. Second individual with visible fungal infection (Paa22) developed symptoms after being held in captivity for 1 month. Besides dense, fungal cotton-like overgrowth on the hind limb, resembling a boot (Figure 4C), the specimen exhibited impaired swimming ability. The limb of the specimen was primarily colonized by several strains of the opportunistic pathogenic oomycete *Saprolegnia parasitica*, and additionally by *Aspergillus creber, A. sydowii, Chalara holubovae, Gnomoniopsis* sp. (nov.), *Peniophora pithya* and *Rutstroemia conformata.* None of the latter three fungal species were isolated from the specimen Paa23 with prominent edema (see above), which was caught at the same locality. Specimen Paa25, held in captivity for 1 month, was infected as well with *Saprolegnia parasitica*, with distinctive hyphal overgrowth on its head and limbs. The last specimen (Paa26) developed moldy overgrowth all over its body after being held in captivity for several years. Fungi isolated from the overgrowth were *Acremonium* sp. (nov.) and *Candida vartiovaarae.*

(iii) The third group of symptomatic animals included two lethargic specimens without other visible pathologies. The first specimen (Paa3) was kept in captivity for over 10 years. It gradually became lethargic and ceased to feed resulting in weight loss. No fungal isolates were obtained from the skin surface of this animal. Second animal with lethargy as the most distinctive symptom (Paa42) has been brought to the cave laboratory, which serves as an asylum for olms washed out of their cave habitat, and only *Aspergillus jensenii* was isolated from this specimen.

**FIGURE 5:**
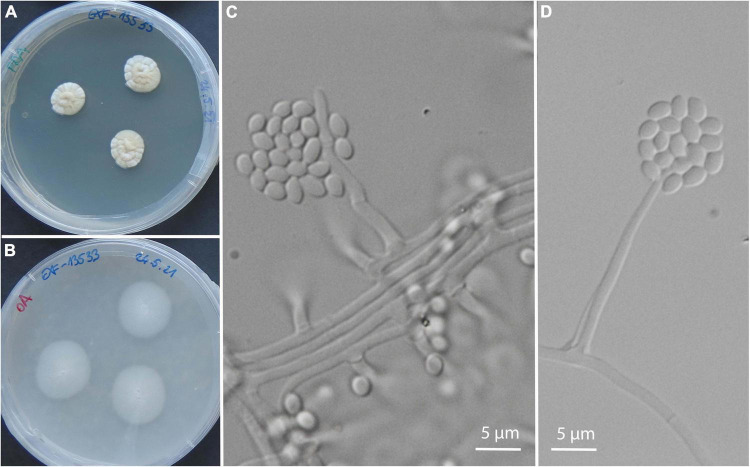
A new species of the genus *Acremonium* isolated from olms in captivity: from several asymptomatic and from a single symptomatic animal. Culture of EXF-13533 on PDA **(A)**, on OA **(B)**; micromorphology of the strain EXF-14260 on PDA **(C,D)**.

Isolates exclusively from symptomatic animals belong to 24 genera and 27 species, most of them with only 1 or 2 isolates: *Acremonium* sp. (nov.) (Figure 5), *Aspergillus sydowii, Cadophora ramosa, Chalara holubovae, Cladosporium halotolerans, Cl. pulvericola, Colpoma* sp. (nov.), *Cystobasidium minutum, Gnomoniopsis* sp. (nov.), *Juxtiphoma eupyrena, Paracremonium* sp. (nov.), *Paracremonium variiforme, Paraphoma* sp. (nov.), *Penicillium crustosum, P. expansum, Peniophora pithya, Phialocephala glacialis, Rutstroemia conformata, Sistotrema brinkmannii, Sydowia polyspora, Talaromyces kabodanensis, Trichoderma citrinum, Truncatella angustata, Xylodon flaviporus*, and unidentified isolates belonging to *Phanerochaete*, Pleosporales, and Helotiales. *Plectosphaerella plurivora* was represented by three isolates from an olm specimen (Paa23), and one additional isolate from environmental water (Supplementary Table 2). The only exception with a higher abundance was the oomycete *Saprolegnia parasitica* with seven isolates from two animals (Table 2), sampled at two different localities, with approximately 3 weeks between the samplings.

The overlap between symptomatic animals in captivity and in nature consisted of one species only: *Cladosporium allicinum.* There were only eight fungal species retrieved from symptomatic or asymptomatic, and/or in nature or in captivity: *A. creber*, *Cl. neolangeronii*, *Cl. pseudocladosporioides*, *Debaryomyces hansenii*, *Parengyodontium album*, *P. brevicompactum*, *P. chrysogenum*, and *T. simmonsii* (Table 2).

### Fungal Diversity From Water and Baits

Water was sampled in two locations (Jelševnik, Planinska jama), while baits were placed in water at four locations (Jelševnik, Krška jama, Planinska jama, Kompoljska jama).

**FIGURE 6:**
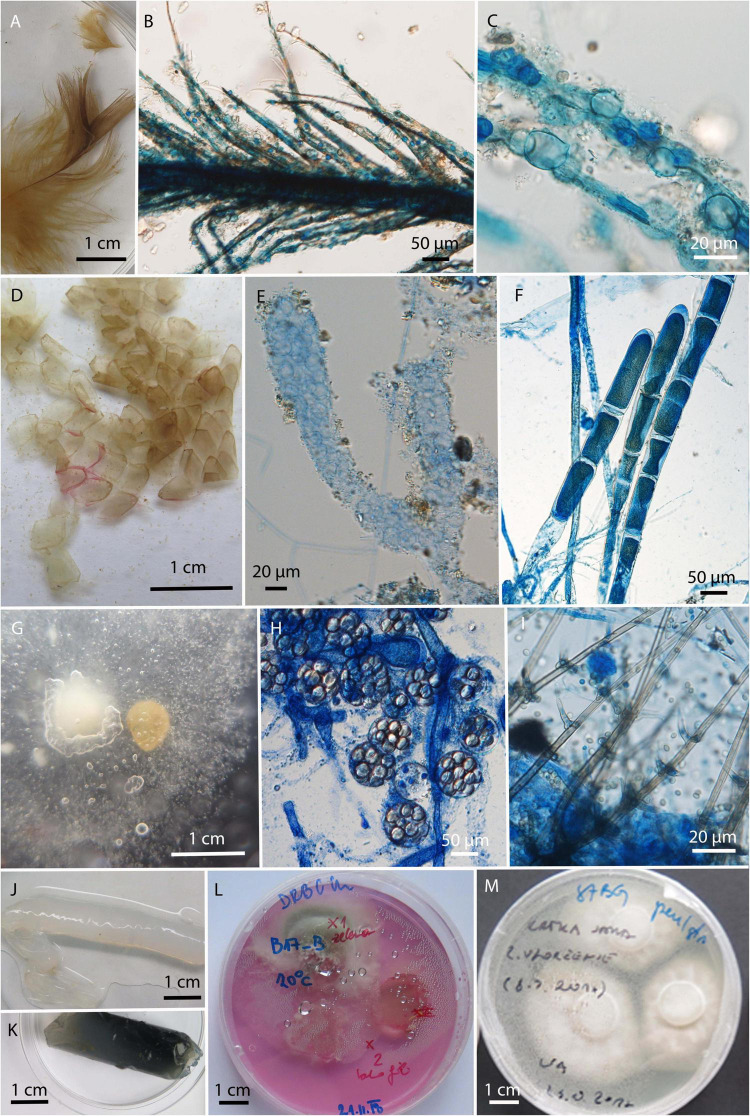
Microbiological baits after incubation in the environmental water. **(A–C)** Bird feathers colonized by unknown chytrid; **(D)** molten snake skin; **(E–F)** unknown filamentous organism growing on molten snake skin; **(G)** canopy seeds after incubation, overgrown with **(H)** *Saprolegnia* () and **(I)** *Chloridium aseptatum*; **(J,K)** dialysis tubes filled with water agar; **(L,M)** cut pieces of agar after incubation on isolation culture media.

In total 129 strains were isolated exclusively from water and baits and were not retrieved from swab samples of animals. They were represented by 22 genera and 52 species. The dominant genera were *Mucor* with six different species and a total of 43 isolates, primarily due to 34 isolates of *Mucor laxorrhizus*, and *Trichoderma* with 12 species and 43 isolates. The diversity of fungi obtained by baiting with keratin, hemp seeds and water agar in dialysis tubes was much higher than obtained by water filtration, mostly due to the overgrowth of fast spreading fungi, like *Trichoderma*, *Fusarium*, and *Mucor* in the latter case (Supplementary Table 3). Some fungi observed on the baits by direct microscopy could not be isolated into pure culture (Figure 6). For example, a chytrid fungus (Figures 6B,C) was only grown on feathers, and mycelia of unidentified, presumably oomycete, from molten snake skin bait (Figures 6E,F).

### Statistical Evaluation of Results

Since most isolated fungi appeared only in individual samples, statistical analyses were performed based on the presence/absence data of individual genera and not individual species.

Statistically significant differences in the presence of fungal genera were observed between different types of samples (ANOVA *p* < 0.01), and the significant difference was confirmed with a *post-hoc* estimated marginal means pairwise comparison between animal swabs and microbial baits (*p* < 0.01) and also between water and baits (*p* < 0.05).

Among animal isolates we observed a statistically significant co-occurrence of the genera *Penicillium* and *Debaryomyces* (Supplementary Figure 1). When considering all samples (animals, baits, and water) the genus *Trichoderma* co-occurred with *Clonostachys*, *Fusarium*, *Mortierella*, and *Mucor*, while *Penicillium* additionally co-occurred with *Pseudogymnoascus*.

Differences in the presence of fungal genera between animals in captivity and nature and differences between symptomatic and healthy animals were not statistically significant (ANOVA *p* > 0.05).

**FIGURE 7:**
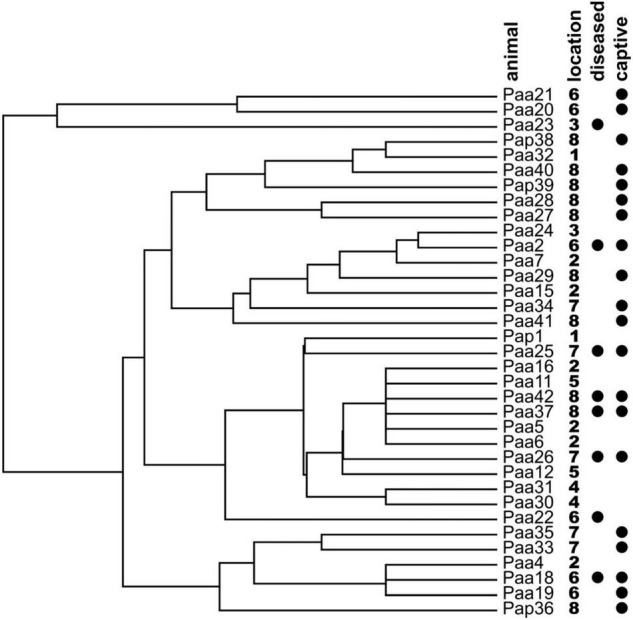
Hierarchical clustering analysis of the olm specimens based on the presence/absence of cultivable fungal genera on the skin of the sampled animals. Locations of specimen samplings are given next to the names of specimen (1: Vivarium 1; 2: crna jama; 3: Vivarium 3; 4: Jelševnik; 5: Planinska jama; 6: Vivarium 2; 7: Vir pri Sticni; and 8: Kompoljska jama). Symptomatic diseased animals and animals held in captivity prior to sampling are marked with black circles.

Also, in hierarchical clustering analysis, the samples from symptomatic or captive samples failed to cluster together (Figure 7).

## Discussion

### Olm’s Opportunistic Fungal Pathogens

The olm is an endemic cave amphibian with a number of extraordinary and unique characteristics. Understanding the interactions of the olm with its environment, including interactions with microbes, is essential for its successful protection. The main goal of this study was to identify potential fungal pathogens of olms and to contribute to a little-known topic for amphibians in general. There are few reports of fungal diseases in wild and captive amphibians, and the causal relationships with clinical signs of infection are often vague, as confirmed with symptoms observed in olms. Symptoms of fungal infection in amphibians include weight loss, ulcers, or multiple granulomas in the skin, skeletal muscles, meninges, and bone marrow (Cicmanec et al., 1973; Taylor et al., 2001). In addition, swelling and lesions of internal organs may occur with visible hyphae and sclerotic bodies in the spleen, liver, kidney, heart, and lungs. Death usually occurs 1–6 months after the first signs of infection. These symptoms were not observed in olms. Zygomycosis, primarily due to infections with *Mucor* spp. (Frank, 1976; Fowler, 1986; Speare et al., 1994; Taylor et al., 1999; Perpinan et al., 2010) and *Rhizopus* spp. (Taylor et al., 1999), can occur in a systemic form (Speare et al., 1994) or as external dermatitis (Taylor et al., 1999). Clinical signs include lethargy and multifocal hyperemic nodules with visible fungal growth, that is in general progressive and leads to death within 2 weeks (Taylor et al., 1999). Although lethargy was an observed symptom in two olms, both in long term captivity, no zygomycosis was determined and only three fungal species were isolated: human opportunistic pathogen *A. jensenii* (Zen Siqueira et al., 2016), plant pathogenic psychrotolerant *P. expansum* (Al Riachy et al., 2021) and soil and plant litter associated saprophytic *Trichoderma simmonsii* (Chaverri et al., 2015). Similar symptoms can occur in cutaneous and disseminated systemic chromomycosis, and phaeohyphomycosis (Seyedmousavi et al., 2013), caused by different pigmented ascomycetous species of the genera *Cladosporium*, *Exophiala*, *Fonsecaea*, *Ochroconis*, *Phialophora*, *Rhinocladiella* and *Scolecobasidium* (Juopperi et al., 2002; Seyedmousavi et al., 2013), which have been isolated from various wild and captive anurans (Dhaliwal and Griffiths, 1963; Elkan and Philpot, 1973; Rush et al., 1974; Beneke, 1978; Miller et al., 1992; Seyedmousavi et al., 2013). Although some of the above listed fungi were isolated from symptomatic olms, they did not cause chromomycosis, and phaeohyphomycosis. If information on fungal diseases of toads and frogs are few, information on fungal diseases of salamanders are even scarcer. In addition to reports of *B. salamandrivorans* infections, only Nickerson et al. (2011) have reported fungal infections in a giant aquatic ozark hellbender salamander (*Cryptobranchus alleganiensis bishopi*). Intact skin was infected with fungal species of the genera *Aureobasidium*, *Cladosporium*, and *Penicillium*, while injured skin sites were colonized with species of the genera *Acremonium*, *Aspergillus*, *Cladosporium*, *Curvularia*, *Exophiala*, *Fusarium*, *Penicillium*, *Sporothrix*, and streptomycetes. With the exception of three reports of saprolegniosis and *Exophiala* infection of olms in captivity, there were so far no data in the literature about fungal infections in cave salamanders (Kogej, 1999; Bizjak-Mali et al., 2018; Lukač et al., 2019).

Out of nine symptomatic olms sampled in this study, only one was from the wild. The time in captivity ranged from only 1 month and up to more than 10 years. The symptomatic olms from captivity were divided into three groups. First, two lethargic specimens described above. Extensive edema was the prevailing symptom in the second group, that included two animals in long- term captivity and the only symptomatic animal from the wild. Although isolates comprised many different fungal species, only the human opportunistic pathogen *C. allicinum* (Sandoval-Denis et al., 2016) was isolated from two of them. From the third specimen only *Lecanicillium coprophilum* was recovered. Species in *Lecanicillium* are known pathogens of arthropods, nematodes, and other fungi (Su et al., 2019). The third group included specimens with visible fungal growth. In the first case (lethargic specimens), diverse fungi, probably representing secondary infectious agents, were isolated from a traumatic injury. The second specimen, which had been in captivity for several years, was moldy all over its body. The dominant isolate likely represents a new species of the genus *Acremonium* which is most similar to *Acremonium sclerotigenum* group according to ITS rDNA sequences (92% similarity). However, the same *Acremonium* species was also detected on skin of asymptomatic animals exclusively in captivity, at two different locations. Other two olm specimens kept in captivity for only 1 month, developed cotton-like overgrowth which was identified as *Saprolegnia parasitica* in one case and as *Cunninghamella* in the other case. Oomycete water molds *Saprolegnia ferax*, *S. parasitica* or *Aphanomyces* spp. are known to cause saprolegniasis in amphibians (Densmore and Green, 2007). Species of *Saprolegnia* are primary skin or oral pathogens in larval amphibians and causative agents of secondary superficial infections in aquatic anurans and urodelans (Kiesecker et al., 2001; Densmore and Green, 2007). These oomycetes can also have a significant impact on amphibian fecundity as well as on egg mortality (Blaustein et al., 1994). Clinical signs include external appearance of mycelium with cotton-like texture, visible hyphae and zoospores in lesions, erythematous or ulcerated skin on tail, hind limbs, gills, and oral mucosa, but rarely with deep tissues lesions (Densmore and Green, 2007). Additional symptoms may include weight loss, lethargy, vomiting, and respiratory distress (Frye and Gillespie, 1989). Previously, there were two reports of infection with *Saprolegnia* spp. on the limbs, gills and even disseminated in other body parts of captive olm specimens (Kogej, 1999; Lukač et al., 2019). It should be noted that the olm has a thin larval skin that is particularly susceptible to infection (Lindinger, 1984). The epidermis of the olm consists of a stratified squamous epithelium covered with mucus, while keratinized skin is present only on the feet (Lindinger, 1984; Bulog, 1994).

### Mycoflora of Asymptomatic Olm Specimens

Since olm has an important ecological role as top predator of underground water systems, its presence reflects the stability of food chains in karst underground water, a unique ecosystem with the highest underground biodiversity in the World. In this light, the health of natural populations of olms may reflect disturbances in their environment such as pollution of water sources that could represent a serious threat to endemic biodiversity and a risk to human health. A study of bacterial diversity of olm skin and their groundwater environment revealed that the identified bacterial taxa were a strongly filtered subset of the environmental microbial community. It was hypothesized that this microbiome protects against invading microbes by competitive exclusion, and thus could serve as an indicator of health status (Kostanjšek et al., 2019). In this light we investigated the diversity of cultivable fungi obtained by swab sampling of asymptomatic specimens both in the wild and in captivity. In accordance, asymptomatic olms from nature resulted in isolation of sporadic colonies, displaying the lowest diversity of fungi in general and few opportunistic species. Although only a small number of fungal isolates were obtained, these isolates differed from specimen to specimen. Among them, the identified genera *Bjerkandera*, *Cystofilobasidium*, *Exophiala, Fusarium, Penicillium, Pseudogymnoascus, Stereum*, *Tausonia*, and *Trametes*, were previously reported from cave environments around the globe (Vanderfolf et al., 2013; Novak-Babič et al., 2016, 2017).

In comparison, the total number of isolates and diversity of fungi isolated from asymptomatic captive animals was considerably higher. Among the fungal genera previously reported from cave environments were *Aspergillus, Barnettozyma, Candida, Cladosporium, Debaryomyces, Exophiala, Mortierella, Mucor, Naganishia*, *Penicillium, Phoma, Pseudogymnoascus, Sporobolomyces*, and *Trichoderma* (Grishkan et al., 2004; Vanderfolf et al., 2013; Novak-Babič et al., 2016, 2017). In addition to these, representatives of 23 fungal genera were isolated that, to the best of our knowledge, had not previously been reported from underground water habitats. Their presence may be related to the human presence and water quality (e.g., *Candida*) or to the materials used in vivaria, e.g., wood related to basidiomycetous genera *Bjerkandera, Byssomerulius, Clitocybe, Peniophora*, and *Phanerochaete* (Boddy et al., 2008; Kirk et al., 2008). Interestingly, the number of yeasts on captive animals was considerably higher than those reported from cave waters, among them *Apiotrichum laibachii*, *A. porosum, A. akiyoshidainum, Barnettozyma californica, Candida friedrichii*, *C. glaebosa, C. saitoana, C. sake*, *C. vartiovaarae, Cutaneotrichosporon cutaneum, Cu. dermatis*, *Cyphellophora olivacea*, *Debaryomyces vindobonensis*, *Dipodascus geotrichum*, *Exophiala alcalophila*, *E. castellanii*, *Naganishia* sp., *Rhodosporidiobolus fluvialis*, and *Sporobolomyces ruberrimus.*

### Subterranean Water Environment as Vector for Amphibian Opportunistic Pathogenic Fungi

Water is crucial for the amphibian life cycle and is one of the main vectors for pathogen transmission. *Batrachochytrium dendrobatidis* and *B. salamandrivorans*, species of *Saprolegnia*, *Cladosporium*, *Mucor*, *Fusarium* and melanized fungi, such as species of genera *Exophiala*, *Rhinocladiella*, *Phialophora* and *Aureobasidium melanogenum* are present in various aquatic environments worldwide (Kiesecker et al., 2001; Göttlich et al., 2002; de Hoog et al., 2011; Defra, 2011; Novak-Babič et al., 2016). In addition to water, amphibians can also become infected through the soil or dead plant matter after stress or traumatic injury of the skin, by ingestion, or by inhalation of fungal spores that can be transported into caves by numerous organisms, including invertebrates, bats, rodents, other animals and humans, as well as by air circulation (Whitaker and Jaw, 2021). In the northern hemisphere, where most cave mycological studies have been conducted, caves are generally characterized by a lack of organic substrates and stable temperatures. This environment favors communities of oligotrophic, psychrotolerant fungi (Vanderfolf et al., 2013). Fungi isolated from asymptomatic animals in the wild reflect this ecology. In this study, Basidiomycota isolates, often associated with nutrient rich substrates such as wood and dung, represented only 8.6% (6 strains of 70) and 1.2% (3 strains of 155) of isolates from animals in nature and captivity, respectively, and only 2.6% (4 strains of 152) of isolates from cave water. Amongst the genera and species most frequently reported in studies on cave mycology (Vanderfolf et al., 2013), we identified the genera *Aspergillus* (3 isolates), *Chaetomium* (1), *Cladosporium* (5), *Fusarium* (11), *Mortierella* (6), *Mucor* (50), *Penicillium* (3), *Trichoderma* (51), and species *Penicillium brevicompactum* (1) and *Trichoderma viride* (2), from both undergound waters and asymptomatic animals. Since the most commonly reported cave fungal taxa were likely influenced by the cultivation media and conditions, rather than biological patterns in cave mycology, we tried to diminish this bias by using four different media and two incubation temperatures (15 and 20°C). This approach resulted in isolation of many rare and unusual taxa, with very low abundance and with 15 taxa that are most probably new for science. As we did not see any pattern of the diversity of commensal fungi isolated from asymptomatic animals from the wild or from captivity, we can conclude that olm in nature is considerably resistant to fungi, with many different species of fungi isolated as single isolates from asymptomatic animals.

## Conclusion

The European blind cave salamander *Proteus anguinus* is a charismatic endemic amphibian that lives in the subterranean waters of the Dinaric Karst. Despite its exceptional conservation importance, not much is known about its ecology and interactions with the groundwater microbiome. The cutaneous microbiota of amphibians is an important driver of metabolic capabilities and immunity, and thus a key factor in their well-being and survival. In this study, we identified a high diversity of fungi to which olm’s skin is exposed in nature and in captivity, reflecting the environmental status of the groundwater. In addition, this study provided the first insight into the presence of opportunistic fungi that threaten proteus in its natural habitat and could represent a health threat not only to proteus, but also to other endangered neotenic salamanders and amphibians inhabiting surface karstic waters. These results provide an initial step toward a comprehensive evaluation and mitigation of fungal threats to this unique amphibian species, which is an important component of its much-needed conservation plan.

## Data Availability Statement

The datasets presented in this study can be found in online repositories. The names of the repository/repositories and accession number(s) can be found in the article/Supplementary Material.

## Ethics Statement

Animals were collected under permit 35601-27/2021-8 issued by Ministry of the Environment and Spatial Planning of the Republic of Slovenia and Slovenian Agency for the Environment.

## Author Contributions

NG-C: conceptualization. PZ, CG, RK, and LB-M: methodology. PZ, AG, RK, and LB-M: investigation. NG-C and RK: resources. PZ and AG: data curation. NG-C, RK, and PZ: writing – original draft preparation. PZ and NG-C: writing – review and editing. RK and PZ: visualization. NG-C and RK: funding acquisition. All authors have read and agreed to the published version of the manuscript.

## Conflict of Interest

The authors declare that the research was conducted in the absence of any commercial or financial relationships that could be construed as a potential conflict of interest.

## Publisher’s Note

All claims expressed in this article are solely those of the authors and do not necessarily represent those of their affiliated organizations, or those of the publisher, the editors and the reviewers. Any product that may be evaluated in this article, or claim that may be made by its manufacturer, is not guaranteed or endorsed by the publisher.

## Footnotes

1
http://www.edgeofexistence.org/species/olm/

2
http://www.ex-genebank.com/
