# Supplementary material for: Cultivable Skin Mycobiota of Healthy and Diseased Blind Cave Salamander (Proteus anguinus)
Source: Front Microbiol. 2022 Jul 14;13:926558. doi: 10.3389/fmicb.2022.926558 (PMC9329069; doi:10.3389/fmicb.2022.926558)
Supplement: Supplementary file 1 [file Data_Sheet_1.docx]

Supplementary Material

# Supplementary Figures and Tables

## Supplementary Figures


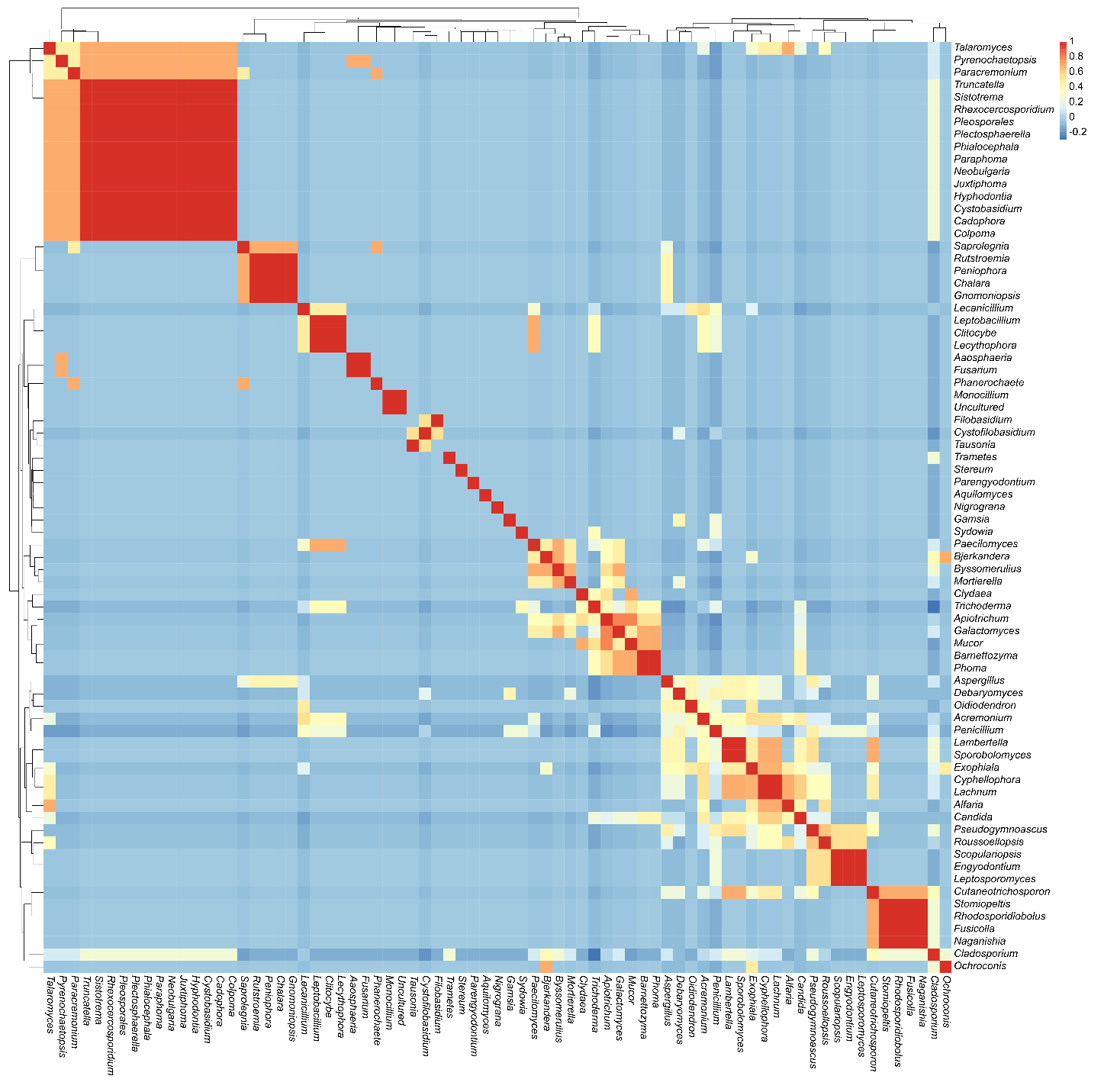


**Supplementary Figure 1.** Co-occurrence of fungal species on *Proteus anguinus* skin.

## Supplementary Tables

**Supplementary Table 1**. Sampled animals, sampling locations and sampling dates.

| **Animal** | **Sampling site** | **Date** | **Animal condition (A, S*)** |
| --- | --- | --- | --- |
| Pap1 | Jelševnik | 28.07.2017 | A |
| Paa2 | Vivarium 1 | 01.08.2017 | S |
| Paa3 | Vivarium 1 | 06.07.2017 | S |
| Paa4 | Planinska jama | 22.08.2017 | A |
| Paa5 | Planinska jama | 22.08.2017 | A |
| Paa6 | Planinska jama | 22.08.2017 | A |
| Paa7 | Planinska jama | 22.08.2017 | A |
| Paa8 | Planinska jama | 22.08.2017 | A |
| Paa9 | Planinska jama | 22.08.2017 | A |
| Paa10 | Planinska jama | 22.08.2017 | A |
| Paa11 | Vir pri Stični | 18.12.2017 | A |
| Paa12 | Vir pri Stični | 18.12.2017 | A |
| Paa13 | Vir pri Stični | 18.12.2017 | A |
| Paa14 | Vir pri Stični | 18.12.2017 | A |
| Paa15 | Planinska jama | 08.03.2018 | A |
| Paa16 | Planinska jama | 08.03.2018 | A |
| Paa17 | Črna jama | 03.04.2018 | A |
| Paa18 | Vivarium 1 | 04.04.2018 | S |
| Paa19 | Vivarium 1 | 16.04.2018 | A |
| Paa20 | Vivarium 1 | 16.04.2018 | A |
| Paa21 | Vivarium 1 | 16.04.2018 | A |
| Paa22 | Vivarium 1 | 03.04.2018 | S |
| Paa23 | Črna jama | 08.05.2018 | S |
| Paa24 | Črna jama | 08.05.2018 | A |
| Paa25 | Vivarium 2 | 26.04.2018 | S |
| Paa26 | Vivarium 2 | 17.06.2015 | S |
| Paa27 | Vivarium 3 | 19.07.2018 | A |
| Paa28 | Vivarium 3 | 19.07.2018 | A |
| Paa29 | Vivarium 3 | 20.07.2018 | A |
| Paa30 | Kompoljska jama | 05.10.2018 | A |
| Paa31 | Kompoljska jama | 05.10.2018 | A |
| Pap32 | Jelševnik | 05.10.2018 | A |
| Paa33 | Vivarium 2 | 23.01.2016 | A |
| Paa34 | Vivarium 2 | 22.08.2017 | A |
| Paa35 | Vivarium 2 | 17.09.2018 | A |
| Pap36 | Vivarium 3 | 05.12.2018 | A |
| Paa37 | Vivarium 3 | 05.12.2018 | S |
| Pap38 | Vivarium 3 | 05.12.2018 | A |
| Pap39 | Vivarium 3 | 05.12.2018 | A |
| Pap40 | Vivarium 3 | 05.12.2018 | A |
| Pap41 | Vivarium 3 | 05.12.2018 | A |
| Pap42 | Vivarium 3 | 01.11.2019 | S |

*Legend: A = asymptomatic animals, S = animals with disease symptoms

**Supplementary Table 2**: List of fungal species isolated from all sampled animals and from environmental samples (water in Krška jama and sporadic water samples from other caves and microbial baits). Legend: asymptomatic animals (A), symptomatic animals (S), natural cave environment (N), captivity (C), number of olm specimens from which fungal strains were isolated (P), water (W), baits (B), total number of isolates of the species (I).

| Taxon Name (identity to the closest type species) | A | S | N | C | P | W | B | I | Representatitive strain | GenBank numbers: ITS rDNA, *BenA*, *act*, EF-1a |
| --- | --- | --- | --- | --- | --- | --- | --- | --- | --- | --- |
| *Aaosphaeria arxii** | + |  | + |  | 1 (Pap1) |  |  | 2 | EXF-12652 | ON312993, /, / |
| *Acremonium* sp. 1* (91% id. *A. alternatum* T) | + | + |  | + | 6 |  |  | 10 | EXF-13533 | ON261226, /, / |
| *Acremonium* sp. 2*** (96% id. *A. persicinum* T) | + |  |  | + | 1 (Paa20) |  |  | 1 | EXF-13547 | ON261225, /, / |
| Hypocreales* (88% id. *Aflaria caricicola* T) | + |  |  | + | 1 (Paa21) |  |  | 1 | EXF-13555 | ON261227, /, / |
| *Apiotrichum akiyoshidainum***^, Y^ | + |  |  | + | 1 (Paa35) |  |  | 1 | EXF-14057 | ON261229, /, / |
| *Apiotrichum laibachii***^, Y^ | + |  |  | + | 3 |  |  | 7 | EXF-14044 | ON261230, /, / |
| *Apiotrichum porosum***^, Y^ | + |  |  | + | 1 (Paa35) |  |  | 4 | EXF-14154 | ON261228, /, / |
| *Aquilomyces* sp.* (86% id. *A. patris* T) | + |  | + |  | 1 (Paa6) |  |  | 1 | EXF-12834 | ON261231, /, / |
| *Aspergillus* *creber** | + | + | + | + | 2 |  |  | 3 | EXF-13522 | ON261233, ON804234, / |
| *Aspergillus jensenii** | + | + |  | + | 2 |  |  | 4 | EXF-13725 | ON261234, /, / |
| *Aspergillus sydowii** |  | + | + |  | 1 (**Paa22**) |  |  | 1 | EXF-13518 | ON261232, ON777800, / |
| *Barnettozyma californica**^, Y^ | + |  |  | + | 1 (Paa35) |  |  | 1 | EXF-14058 | ON261236, /, / |
| *Bjerkandera adusta*** | + |  | + | + | 2 |  |  | 2 | EXF-14270 | ON261237, /, / |
| *Byssomerulius corium*** | + |  |  | + | 1 (Paa41) |  |  | 1 | EXF-14269 | ON312992, /, / |
| *Cadophora ramosa** |  | + | + |  | 1 (**Paa23**) |  |  | 1 | EXF-13528 | ON261238, /, / |
| *Candida friedrichii**^, Y^ | + |  |  | + | 1 (Paa20) |  |  | 1 | EXF-13549 | ON261240, /, / |
| *Candida glaebosa**^, Y^ (98% id. T) | + |  |  | + | 1 (Paa20) |  |  | 1 | EXF-13703 | ON261242, /, / |
| *Candida saitoana**^, Y^ | + |  |  | + | 1 (Paa21) |  |  | 4 | EXF-13550 | ON261241, /, / |
| *Candida sake**^, Y^ | + |  |  | + | 1 (Paa19) |  |  | 3 | EXF-13530 | ON261239, /, / |
| *Candida vartiovaarae**^, Y^ | + | + |  | + | 2 |  |  | 2 | EXF-13828 | ON261243, /, / |
| *Chalara holubovae** |  | + | + |  | 1 (**Paa22**) |  |  | 1 | EXF-13523 | ON261244, /, / |
| *Rutstroemiaceae** (*Ciboria*, *Lambertella* sp. 99% id.) | + |  |  | + | 1 (Paa20) |  |  | 1 | EXF-13537 | ON261245, /, / |
| *Cladosporium allicinum** |  | + | + | + | 2 |  | + | 4 | EXF-13525 | ON261246, /, ON777803 |
| *Cladosporium asperulatum** | + |  |  | + | 1 (Paa28) |  |  | 1 | EXF-13709 | ON261247, /, ON777804 |
| *Cladosporium halotolerans** |  | + |  | + | 1 (**Paa2**) |  |  | 1 | EXF-13221 | ON261248, /, ON777805 |
| *Cladosporium neolangeronii** | + | + | + | + | 3 |  |  | 4 | EXF-13583 | ON261249, /, ON777806 |
| *Cladosporium phyllophilum** | + |  |  | + | 1 (Paa24) |  |  | 1 | EXF-13697 | ON261250, /, ON777807 |
| *Cladosporium pseudocladosporioides** | + | + | + | + | 3 | + |  | 5 | EXF-13382 | ON261251, /, ON777808 |
| *Cladosporium psychrotolerans** | + |  |  | + | 1 (Paa34) |  |  | 1 | EXF-14155 | ON261252, /, / |
| *Cladosporium pulvericola** |  | + |  | + | 1 (**Paa2**) |  |  | 1 | EXF-13219 | ON261253, /, ON804235 |
| *Clitocybe* sp*.*** (100% id. *C. nebularis, robusta*, 91% id. *C.* *brunneocaperata* T) | + |  |  | + | 1 (Pap36) |  |  | 1 | EXF-14804 | ON261254, /, / |
| *Colpoma* sp.* (89% id. *C. junipericola* T) |  | + | + |  | 1 (**Paa23**) |  |  | 1 | EXF-13656 | ON261255, /, / |
| Coniochaetaceae*** (*Vexillomyces/Claussenomyces/Sclerotinia* 99% id.) | + |  |  | + | 1 (Pap36) |  |  | 1 | EXF-14805 | ON261256, /, / |
| *Cutaneotrichosporon cutaneum***^, Y^ | + |  |  | + | 1 (Paa20) |  |  | 1 | EXF-13539 | ON261257, /, / |
| *Cutaneotrichosporon dermatis***, ^Y^ | + |  |  | + | 1 (Paa34) |  |  | 2 | EXF-14043 | ON261258, /, / |
| Cylindrosympodiaceae*** (*Cylindrosympodium, Tothia, Pseudoanungitea*) | + |  |  | + | 1 (Paa34) |  |  | 1 | EXF-14041 | ON261259, /, / |
| *Cyphellophora olivacea**^, BY^ | + |  |  | + | 2 |  |  | 5 | EXF-13558 | ON261260, /, / |
| *Cystobasidium minutum***^, Y^ |  | + | + |  | 1 (**Paa23**) |  |  | 2 | EXF-13608 | ON261261, /, / |
| *Cystofilobasidium* sp.**^, Y^ (95% *id. C. macerans* T) | + |  | + |  | 3 |  |  | 6 | EXF-13829 | ON261262, /, / |
| *Debaryomyces vindobonensis**^, Y^ | + |  |  | + | 1 (Pap40) |  |  | 1 | EXF-14255 | ON261264, /, / |
| *Debaryomyces hansenii**^, Y^ | + | + | + | + | 4 |  |  | 9 | EXF-13548 | ON261263, /, / |
| *Dipodascus geotrichum**^, Y^ | + |  |  | + | 2 |  |  | 6 | EXF-14047 | ON261265, /, / |
| *Exophiala alcalophila**^, BY^ | + |  |  | + | 1 (Pap39) |  |  | 2 | EXF-14261 | ON261266, /, / |
| *Exophiala* *castellanii**^, BY^ | + |  |  | + | 2 |  |  | 3 | EXF-13556 | ON261267, /, / |
| *Exophiala lecanii-corni**^, BY^ | + |  | + |  | 1 (Paa15) |  |  | 1 | EXF-13667 | ON261268, /, / |
| *Filobasidium stepposum**^, y^* | + |  | + |  | 1 (Paa30) |  |  | 1 | EXF-13826 | ON261269, /, / |
| *Fusarium sporotrichioides** | + |  | + |  | 1 (Pap1) | + | + | 3 | EXF-12901 | ON261270, /, / |
| *Fusicolla aquaeductuum** | + |  |  | + | 1 (Paa34) |  |  | 1 | EXF-14505 | ON261271, /, / |
| *Gamsia* sp*.** (96% id. *G. columbina* T) | + |  |  | + | 1 (Pap38) |  |  | 1 | EXF-14245 | ON261272, /, / |
| *Gnomoniopsis* sp.* (97% id. G. *racemula, alderdunensis* T) |  | + | + |  | 1 (**Paa22**) |  |  | 1 | EXF-14812 | ON261273, /, / |
| *Helotiales* sp. 1* (97% id. *Cadophora luteo-olivacea* T, 97% id. *Mycochaetophora gentianae* T, 97% id. *Rhexocercosporidium senecioni*s T) | + |  | + |  | 1 (Paa12) |  |  | 1 | EXF-13262 | ON312994, /, / |
| *Helotiales* sp. 2* (91% id. *Piniphoma wesendahlina* T) |  | + | + |  | 1 (**Paa23**) |  |  | 1 | EXF-13657 | ON261311, /, / |
| *Juxtiphoma eupyrena** |  | + | + |  | 1 (**Paa23**) |  |  | 1 | EXF-13561 | ON261274, /, / |
| *Lachnum controversum** | + |  |  | + | 1 (Paa21) |  |  | 1 | EXF-13664 | ON261276, /, / |
| *Lachnum pudibundum** | + |  |  | + | 1 (Paa20) |  |  | 1 | EXF-13546 | ON261275, /, / |
| *Lecanicillium coprophilum** | + | + |  | + | 4 |  |  | 5 | EXF-14241 | ON261277, /, / |
| *Leptobacillium leptobactrum** | + |  |  | + | 1 (Pap36) |  |  | 1 | EXF-14237 | ON261278, /, / |
| *Leptosporomyces* sp.**** (97% id. *L.* *fuscostratus*) | + |  |  | + | 1 (Paa27) |  |  | 1 | EXF-14149 | ON261279, /, / |
| *Mortierella alpina**** | + |  |  | + | 1 (Paa40) |  | + | 3 | EXF-14504 | ON261280, /, / |
| *Linnemannia amoeboidea**** | + |  |  | + | 1 (Paa41) |  |  | 1 | EXF-14271 | ON261281, /, / |
| *Mucor circinelloides**** | + |  |  | + | 2 |  | + | 9 | EXF-14036 | ON261282, /, / |
| *Mucor racemosus**** | + |  |  | + | 2 |  |  | 2 | EXF-14056 | ON261283, /, / |
| *Naganishia* sp.**^, Y^ (99% id. *N. diffluens, albidiosimilis* T) | + |  |  | + | 1 (Paa34) |  |  | 1 | EXF-14042 | ON261284, /, / |
| *Niesslia tenuis** | + |  | + |  | 1 (Paa12) |  |  | 2 | EXF-13214 | ON261285, /, / |
| *Nigrograna* sp.*** (97% id. *N. norvegica* T) | + |  | + |  | 1 (Paa5) |  |  | 1 | EXF-12833 | ON261286, /, / |
| *Scolecobasidium globale** | + |  | + |  | 1 (Paa15) |  |  | 1 | EXF-13582 | ON261287, /, / |
| *Oidiodendron eucalypti** | + |  |  | + | 1 (Pap39) |  |  | 1 | EXF-14251 | ON261288, /, / |
| *Paracremonium* sp.* (96% *P. inflatum, variiforme* T) |  | + | + |  | 1 (Paa23) |  | + | 1 | EXF-13663 | ON261289, /, / |
| *Paracremonium variiforme** |  | + | + |  | 1 (**Paa25**) |  |  | 2 | EXF-14503 | ON261290, /, / |
| *Paraphoma* sp.*** (93% id. *P.* *salicis* T) |  | + | + |  | 1 (**Paa23**) |  |  | 1 | EXF-13705 | ON261291, /, / |
| *Parengyodontium album** | + | + | + | + | 2 |  |  | 4 | EXF-13216 | ON261292, /, / |
| *Penicillium atrosanguineum** | + |  |  | + | 2 |  |  | 2 | EXF-14265 | ON261293, ON777794, / |
| *Penicillium bialowiezense** | + |  | + |  | 1 (Pap32) |  |  | 1 | EXF-13836 | ON261294, ON804233, / |
| *Penicillium brevicompactum** | + | + | + | + | 2 |  |  | 3 | EXF-13218 | ON261295, ON777795, / |
| *Penicillium chrysogenum** | + | + | + | + | 5 |  |  | 9 | EXF-13707 | ON261296, ON777793, / |
| *Penicillium citreonigrum** | + |  |  | + | 2 |  |  | 2 | EXF-14264 | ON261297, ON777796, / |
| *Penicillium crustosom** |  | + |  | + | 1 (**Paa2**) |  |  | 1 | EXF-12654 | /, ON777797, / |
| *Penicillium pancosmium** | + |  |  | + | 1 (Pap36) |  |  | 1 | EXF-14239 | /, ON777799, / |
| *Penicillium roseopurpureum** | + |  |  | + | 3 |  |  | 5 | EXF-14263 | ON261300, ON777801, / |
| *Peniophora pithya*** |  | + | + |  | 1 (**Paa22**) |  |  | 1 | EXF-13520 | ON261302, /, / |
| *Phanerochaete* sp.**** (99% id. *P. concrescens* T, 99% id. *P. livescens,* 99% id. *P. sordida*) |  | + | + |  | 1 (**Paa25**) |  |  | 1 | EXF-13668 | ON261303, /, / |
| *Phialocephala* *glacialis** |  | + | + |  | 1 (**Paa23**) |  |  | 1 | EXF-13660 | ON261304, /, / |
| *Neobulgaria koningiana** |  | + | + |  | 1 (**Paa23**) |  |  | 2 | EXF-13526 | ON261305, /, / |
| *Phoma herbarum** | + |  |  | + | 1 (Paa35) |  |  | 1 | EXF-14153 | ON261306, /, / |
| *Plectosphaerella plurivora** |  | + | + |  | 1 (**Paa23**) |  | + | 4 | EXF-13524 | ON261307, /, / |
| *Pleosporales** (91% id. *Piniphoma wesendahlina* T) |  | + | + |  | 1 (**Paa23**) |  |  | 2 | EXF-13659 | ON261308, /, / |
| *Pseudogymnoascus* sp. 1* | + |  |  | + | 3 |  |  | 8 | EXF-13724 | ON261309, /, / |
| *Pseudogymnoascus* sp. 2,3,4* | + |  |  | + | 3 |  |  | 3 | EXF-13720, EXF-13721, EXF-13535 | ON312998, /, /  ON312996, /, /  ON312997, /, / |
| *Pyrenochaetopsis leptospora** | + | + | + |  | 2 |  |  | 3 | EXF-12651 | ON261310, /, / |
| *Rhodosporidiobolus fluvialis***^, Y^ | + |  |  | + | 1 (Paa34) |  |  | 1 | EXF-14040 | ON261312, /, / |
| *Rutstroemia conformata** |  | + | + |  | 1 (**Paa22**) |  |  | 1 | EXF-13519 | ON261313, /, / |
| *Samsoniella hepiali** | + |  |  | + | 2 |  |  | 3 | EXF-14240 | ON261314, /, / |
| *Saprolegnia parasitica***** |  | + | + |  | 2 |  |  | 7 | EXF-13578 | ON261315, /, / |
| *Scopulariopsis brumptii** | + |  |  | + | 1 (Paa27) |  |  | 1 | EXF-13877 | ON261316, /, / |
| *Sistotrema brinkmannii*** |  | + | + |  | 1 (**Paa23**) |  |  | 1 | EXF-13696 | ON261317, /, / |
| *Sporobolomyces ruberrimus***^, Y^ | + |  |  | + | 1 (Paa20) |  |  | 1 | EXF-13532 | ON261318, /, / |
| *Stereum hirsutum*** | + |  | + |  | 1 (Paa16) |  |  | 1 | EXF-13384 | ON261319, /, / |
| *Sydowia polyspora** |  | + |  | + | 1 (**Paa18**) |  |  | 1 | EXF-13584 | ON261320, /, / |
| *Talaromyces rugulosus** | + |  |  | + | 1 (Paa21) |  |  | 1 | EXF-13559 | ON261321, /, / |
| *Talaromyces kabodanensis** |  | + | + |  | 1 (**Paa23**) |  |  | 1 | EXF-13536 | ON261322, /, / |
| *Tausonia pullulans***^, Y^ | + |  | + |  | 1 (Paa31) |  |  | 5 | EXF-13830 | ON261323, /, / |
| *Trametes versicolor*** | + |  | + |  | 1 (Paa7) |  |  | 1 | EXF-12835 | ON261324, /, / |
| *Trichoderma citrinum** |  | + |  | + | 1 (**Paa18**) |  |  | 1 | EXF-13586 | ON261325, /, / |
| *Trichoderma harzianum** | + |  |  | + | 1 (Pap36) |  | + | 5 | EXF-14814 | ON261326, /, / |
| *Trichoderma simmonsii** | + | + | + | + | 2 | + |  | 3 | EXF-12832 | ON261327, /, / |
| *Trichoderma viride** | + |  |  | + | 2 |  | + | 4 | EXF-14038 | ON261328, /, / |
| *Truncatella angustata** |  | + | + |  | 1 (**Paa23**) |  |  | 1 | EXF-13562 | ON261329, /, / |
| *Xylodon flaviporus*** |  | + | + |  | 1 (**Paa23**) |  |  | 1 | EXF-13658 | ON261330, /, / |

Legend: +: isolation of fungal species from a particular source; *‒****: affiliation to the main fungal phyla, *Ascomycota, **Basidiomycota, ***Mucoromycota, **** Oomycota; ^Y^: yeast; ^BY^: black yeast; Paa = *Poteus anguinus anguinus*; Pap = *Proteus anguinus parkelj*. Labels of symptomatic animals are written in bold and underlined.

**Supplementary Table 3**: List of fungal species isolated exclusively from cave water and microbial baits.

|  | Water | Bait  (cf/ss/wa/hs) | Cave | Number of isolates | Representative strain | GenBank numbers: ITS rDNA, *BenA*, EF-1a |
| --- | --- | --- | --- | --- | --- | --- |
| *Acrostalagmus luteoalbus** |  | - /-/+/- | Krška jama | 1 | EXF-12462 | ON312944, /, / |
| *Cadophora melinii** |  | - /-/+/- | Kompoljska jama | 1 | EXF-14112 | ON312945, /, / |
| *Chloridium aseptatum** |  | -/-/-/+ | Jelševnik | 1 | EXF-14808 | ON312946, /, / |
| *Clonostachys rosea** | **+** | -/-/+/- | Jelševnik, Krška jama, Planinska jama | 7 | EXF-12658 | ON312947, /, / |
| *Cylindrodendrum hubeiense** |  | -/+/-/- | Planinska jama | 1 | EXF-14151 | ON312948, /, / |
| *Entyloma randwijkense** |  | -/+/-/- | Krška jama | 1 | EXF-13840 | ON312949, /, / |
| *Fusarium avenaceum** |  | -/-/+/- | Krška jama | 1 | EXF-12656 | ON312950, /, ON804225 |
| *Fusarium culmorum** |  | -/+/-/- | Jelševnik, Planinska jama | 2 | EXF-14026 | ON312951, /, ON804226 |
| *Fusarium elaeagni** | + |  | Jelševnik | 1 | EXF-12660 | ON312952, /, ON804227 |
| *Fusarium nirenbergiae** | + | -/+/-/- | Jelševnik, Planinska jama | 2 | EXF-12659 | ON312953, /, ON804228 |
| *Fusarium* sp.* (100% id. *F.* *acutatum* T*, F. concentricum* T) |  | -/-/+/- | Krška jama, Kompoljska jama | 2 | EXF-13353 | ON312954, /, / |
| *Humicola* sp.*** (100% id. *H. nivea* T*, H. glauca* T*, H. variabilis* T*, H. sardinia* T) |  | -/-/+/- | Jelševnik | 2 | EXF-14507 | ON312955, /, / |
| *Humicola subspiralis** |  | -/-/+/- | Kompoljska jama | 1 | EXF-14469 | ON312956, /, / |
| *Hymenochaetaceae*** |  | -/-/+/- | Krška jama | 1 | EXF-13249 | ON312991, /, / |
| *Linnemannia gamsii**** |  | -/-/**+**/+ | Jelševnik | 2 | EXF-14510 | ON312959, /, / |
| *Mortierella fluviae**** |  | -/-/+/- | Kompoljska jama | 1 | EXF-14098 | ON312957, /, / |
| *Mucor circinelloides**** |  | -/-/+/- | Krška jama | 1 | EXF-14023 | ON312960, /, / |
| *Mucor fragilis**** |  | -/+/-/- | Krška jama | 1 | EXF-13211 | ON312961, /, / |
| *Mucor hiemalis**** |  | **+**/+/-/- | Jelševnik, Kompoljska jama | 2 | EXF-13332 | ON312962, /, / |
| *Mucor laxorrhizus**** |  | +/**+**/+/- | Jelševnik, Krška jama, Planinska jama, Kompoljska jama | 34 | EXF-14092 | ON312963, /, / |
| *Mucor moelleri**** |  | **+**/+/+/- | Jelševnik, Kompoljska jama | 4 | EXF-13333 | ON312964, /, / |
| *Ceriporiopsis gilvescens*** |  | -/+/-/- | Planinska jama | 1 | EXF-13863 | ON312965, /, / |
| *Neopyrenochaeta acicola** |  | +/-/-/- | Planinska jama | 1 | EXF-14150 | ON312966, /, / |
| *Paracremonium binnewijzendii** |  | -/-/+/- | Jelševnik | 2 | EXF-14506 | ON312967, /, / |
| *Penicillium expansum** |  | -/-/+/- | Planinska jama | 1 | EXF-14022 | ON26129, ON777798, / |
| *Penicillium janczewskii** |  | -/-/+/- | Kompoljska jama | 1 | EXF-14075 | ON312968, ON777802, / |
| *Peniophora* sp. 1** (95% id. *P. molesta* T, 99% id. *P. pini*) |  | -/-/+/- | Krška jama | 1 | EXF-13248 | ON312969, /, / |
| *Peniophora* sp. 2** (98% *P. simulans* T, 99% id. *P. quercina*) |  | -/-/+/- | Jelševnik | 1 | EXF-13855 | ON312970, /, / |
| *Pezizomycotina** (90% id. *Phialemoniopsis curvata* T) |  | -/-/+/- | Krška jama | 1 | EXF-12463 | ON312971, /, / |
| *Podila horticola**** |  | -/-/+/- | Jelševnik, Planinska jama | 2 | EXF-14024 | ON312958, /, / |
| *Pythium monospermum***** |  | -/-/+/- | Kompoljska jama | 1 | EXF-14097 | ON312972, /, / |
| *Saprolegnia delica***** |  | -/-/+/- | Krška jama | 1 | EXF-13350 | ON312973, /, / |
| *Saprolegnia* ferax**** |  | -/+/-/- | Planinska jama | 1 | EXF-14512 | ON312974, /, / |
| *Tilletiopsis washingtonensis*** |  | -/-/+/- | Kompoljska jama | 2 | EXF-13865 | ON312975, /, / |
| *Trichoderma atrobrunneum** | + | +/+/**+**/- | Jelševnik, Krška jama, Kompoljska jama | 8 | EXF-13330 | ON312977, /, / |
| *Trichoderma atroviride** |  | -/**+**/+/- | Jelševnik, Krška jama, Planinska jama | 7 | EXF-13347 | ON312978, /, / |
| *Trichoderma citrinoviride** | + |  | Planinska jama | 1 | EXF-12828 | ON312979, /, / |
| *Trichoderma crassum** |  | +/-/+/- | Planinska jama, Kompoljska jama | 2 | EXF-13868 | ON312980, /, / |
| *Trichoderma hamatum** | **+** | +/+/+/- | Jelševnik, Krška jama, Planinska jama | 8 | EXF-12826 | ON312981, /, ON804229 |
| *Trichoderma longibrachiatum** |  | -/+/-/- | Krška jama | 1 | EXF-13207 | ON312982, /, / |
| *Trichoderma paraviridescens** |  | -/-/+/- | Jelševnik, Kompoljska jama | 3 | EXF-14118 | ON312983, /, ON804230 |
| *Trichoderma sichuanense** |  | -/-/+/- | Kompoljska jama | 1 | EXF-14079 | ON312985, /, ON804231 |
| *Trichoderma yunnanense** |  | -/-/+/- | Krška jama | 1 | EXF-13331 | ON312976, /, / |
| *Trichoderma spirale** |  | -/+/-/- | Jelševnik | 1 | EXF-13346 | ON312986, /, / |
| *Trichoderma tomentosum** |  | -/-/+/- | Krška jama | 1 | EXF-12465 | ON312987, /, / |
| *Trichoderma* sp.* (100% id. to different species) | + | -/-/**+**/- | Jelševnik, Planinska jama, Kompoljska jama, Krška jama | 9 | EXF-14078; EXF-14099 | ON312984, /, /; ON312988, /, ON804232 |
| *Volutella* sp. 1* (100% id. to different species) |  | -/-/-/+ | Jelševnik | 1 | EXF-14509 | ON312989, /, / |
| *Volutella* sp. 2* (100% id. to different species) |  | -/-/-/+ | Planinska jama | 1 | EXF-14468 | ON312990, /, / |

Legend: +: isolation of fungal species from a particular source. *‒****: affiliation to the main fungal phyla: *Ascomycota, **Basidiomycota, ***Mucoromycota, **** Oomycota; cf/ss/wa/hs (bait abbreviations): cf = chicken feathers; ss = snake skin; wa = water agar in dialysis tubes; hs = hemp seeds. T=ex-type strains.
